# Influence of Combined Heat Treatment and Hot Isostatic Pressure (HT-HIP) on Titanium Aluminide Processed by L-PBF

**DOI:** 10.3390/ma16145071

**Published:** 2023-07-18

**Authors:** Hatem A. Soliman, James Pineault, Mohamed Elbestawi

**Affiliations:** 1 Department of Mechanical Engineering, McMaster University, 1280 Main Street West, Hamilton, ON L8S 4L7, Canada; elbestaw@mcmaster.ca; 2 Proto Mfg. Ltd., 6150 Morton Industrial Parkway, LaSalle, ON N9J 3W3, Canada; xrdlab@protoxrd.com

**Keywords:** titanium aluminide (TiAl), laser powder bed fusion (L-PBF), heat treatment (HT), hot isostatic pressing (HIP), nanohardness

## Abstract

Postprocessing is essential for improving titanium aluminide (TiAl) microstructure and part quality after using the laser powder bed fusion (L-PBF) method. It has been reported that Ti-48Al-2Cr-2Nb (%at) processed by L-PBF has internal defects and low fracture toughness. Microstructure control by heat treatment (HT) showed a significant improvement in the ductility of the material. Alternatively, hot isostatic pressing (HIPing) could be applied to reduce the residual stresses and internal defects formed during the L-PBF. Combining the benefits of these two subsequent processes into a single predetermined process is appealing for Ti-48Al-2Cr-2Nb (%at) to minimize cost. This work presents a novel strategy to postprocess L-PBF TiAl by applying combined heat treatment and hot isostatic pressing in one process, namely HT-HIP. The process includes three cycles with different conditions (i.e., temperature, time, and pressure). These conditions were determined to achieve improved part quality and microstructure. The results show that the tensile residual stresses decreased from a peak of 249 MPa in the as-built sample to compressive stresses that peaked at −90 MPa after the HT-HIP process. The number and size of internal defects could be greatly reduced. The defects were transformed into a regular spherical shape, which is good in terms of fatigue strength. Additionally, a duplex microstructure with lamellar α_2_/γ colonies could be introduced for better ductility. Different levels of duplex microstructure could be achieved along with the process cycles. The grain structure using EBSD analysis showed refined equiaxed grains, which demonstrate better strength after the HT-HIP process. Twinning boundaries were also observed in the HT-HIP sample. The grain orientation tendency to the build direction significantly reduced after the HT-HIP process. The nanoindentation test was applied to evaluate the nanohardness of the as-built and HT-HIP samples. It could be demonstrated that the nanohardness is dependent on the formed phases and lamellar density inside the grains. The mean hardness value was 8.19 GPa for the as-built sample, while it was 5.48 GPa for the HT-HIP sample.

## 1. Introduction

Titanium aluminide (TiAl), Ti-48Al-2Cr-2Nb (%at), is an advanced intermetallic alloy that is widely used in aerospace industry. The combination of high strength and low density makes it superior over other heavy alloys. Its excellent high-temperature performance has promoted the replacement of nickel superalloys by TiAl in low-pressure turbine blades [1]. However, the high toughness and high crack sensitivity remain inherent issues during the production, machining, and thermoformability of this intermetallic alloy [2]. Recently, significant research has been conducted to overcome these difficulties by using advanced near-net shape manufacturing technology, such as additive manufacturing (AM), and/or by controlling the microstructure using postheat treatment (HT) [3,4]. Despite the success of electron beam melting (EBM) among other AM process to produce crack-free TiAl products, laser powder bed fusion (L-PBF) remains a relevant and compelling technology for TiAl manufacturing due to its process flexibility, improved surface quality, design freedom, and ongoing material/process development [5].

As an advanced near-net shape manufacturing technology, laser powder bed fusion (L-PBF) has shown an efficient capability to produce different metals in complex shapes with high geometrical accuracy [6]. This could significantly reduce the required finishing, machining, and forming processes needed after the traditional processing of brittle materials such as TiAl. Nevertheless, the rapid cooling and high thermal gradient accompanying this technology induce high residual stresses inside the fabricated parts. If the material cannot resist these stresses, the part will fail [7]. Prior research endeavors could mitigate the extent of the residual stresses by introducing different thermal, geometrical, and compositional strategies to reduce the cooling rate during the L-PBF process [8,9,10,11,12]. Additionally, specific investigations have focused on modeling the melt pool and elucidating the impact of process conditions on its physics, thereby enabling control over factors influencing melt pool morphology and the occurrence of associated defects [13]. The sequential thermal scanning (STS) strategy is one of the most recent L-PBF studies that has been introduced to mitigate the internal defects in TiAl parts [14]. The STS strategy utilizes the laser energy to sequentially preheat, melt, and postheat the powder layer using appropriate laser parameters for each scan. The strategy has been demonstrated to significantly increase the part quality and density using lower melting energies, 101.01–138.89 J/mm^3^, compared with the other traditional L-PBF strategies. However, some internal defects such as micropores and/or cracks were observed. Thus, the hot isostatic processing (HIP) was highly recommended [15,16].

The TiAl mechanical properties mainly depend on the processed microstructure and the HT conditions. The solidification route after thermomechanical processing leads to a microstructure dependent on the cooling rate and alloy composition [17,18]. This processed microstructure can vary sensitively by the post-HT. The solidification route of Ti-48Al-2Nb-2Cr (at%) during L-PBF follows the decomposition path of α→α + γ→α_2_ + γ [19]. However, it has been observed that the final microstructure was dominated by α_2_ phase, while the γ phase scarcely existed [11]. This was attributed to the rapid cooling that does not allow the γ phase to sufficiently decompose [20]. The dominance of the α_2_ phase increases the alloy strength at the expense of the ductility [21]. Therefore, the post-HT is necessary to tailor the microstructure for improved mechanical properties. The HTed grain structure, grain size, and phase fraction can control ductility over strength [22]. In the open literature, four HTed microstructures have been reported for TiAl, 36–50 Al (at%), at different annealing temperatures: equiaxed, fully lamellar, nearly lamellar, and duplex microstructure [23]. The equiaxed microstructure is a monophase microstructure with γ grains, which forms at lower annealing temperatures, just above the eutectoid temperature, where the γ volume fraction is high. This microstructure demonstrated high ductility at room temperature but low tensile strength and creep properties. In contrast, the fully and nearly lamellar microstructure are dual-phase microstructures of α_2_ and γ, arranged in lamellar colonies of coarse grains, which forms at higher annealing temperatures, above and just below the α transus line. The fully lamellar microstructure showed higher tensile strength and creep properties but less ductility [24]. The nearly lamellar microstructures have been reported with more balanced strength and ductility than fully lamellar microstructures [25]. The duplex microstructure contains a combination of equiaxed grains and lamellar colonies, which forms at annealing temperatures of 1200–1250 °C, in the α + γ region, where the volume fraction of γ and α are almost equal. The duplex microstructure is usually favored for better ductility and moderate strength. More details can be found about the HT microstructure and phase formation in [26].

Hot isostatic pressing (HIP) has been shown to have a significant effect on eliminating internal defects after L-PBF [27,28,29]. The principles of HIP have been summarized in previous work [30,31]. The main advantage of HIP is to release the induced tensile residual stresses after the L-PBF and introduce compressive stresses. This is thanks to the isostatic pressure applied. Moreover, the high temperature along with the pressure reinforces the closure of pores and microcracks formed in the L-PBF parts. The HIP benefits have been proven in several studies on Ti alloys such as Ti6Al4V. It was demonstrated that HIP can reduce the microstructure heterogeneity attributed to the layer-based L-PBF [32]. In addition, compressed structures with less porosity have been observed [33]. This multiadvantage process is essential for brittle intermetallic materials such as TiAl. Despite the importance of HIP, few studies have been presented in the literature on hot isostatic pressing Ti-48Al-2Nb-2Cr (at%) after L-PBF. Recently, Xiao et al. investigated HIP influence on the microstructure of Ti-48Al-2Nb-2Cr (at%) [34]. Although their study adopted one HIP cycle with a relatively short time of 2 h under 1225 °C and 160 MPa, their experiment could demonstrate that HIP has a beneficial effect on microstructure homogeneity and grain refining. It is worth mentioning that the temperature and pressure values are critical factors in the HIP process. Therefore, it was reported that the operating temperature should be higher than 0.7 of the melting temperature (i.e., 900 to 1150 °C for TiAl). In addition, the applied pressure should be sufficient to cause plastic deformation at this temperature (i.e., 35–200 MPa for TiAl). Equations (1) and (2) present the effective pressures during two levels of densification [35,36]:

(1) $$P_{{eff}_{1}}=\frac{P(1-d_{0})}{d^{2}(d-d_{0})}+\frac{3E_{sp}}{r}d^{2}\left(\frac{2d-d_{0}}{1-d_{0}}\right),\;\mathrm{relative}\;\mathrm{density}\;<0.9$$

(2) $$P_{{eff}_{2}}=P+\frac{2E_{sp}}{r}{\left(\frac{6d}{1-d}\right)}^{1/3}-P_{0}\left(\frac{(1-d_{c})d}{(1-d)d_{c}}\right),\;\mathrm{relative}\;\mathrm{density}\;<0.9$$

where *P_eff_* is the effective pressure, *P* is the external pressure, *P*_0_ is the outgassing pressure, *d* is the relative density, *d*_0_ is the initial relative density, *d_c_* is the relative density at which pores close, *r* is the particle radius, and *E_sp_* is the specific surface energy.

The novelty of this study lies in the proposal of a novel postprocessing strategy, HT-HIP, for laser-melted Ti-48Al-2Nb-2Cr (at%). Ti-48Al-2Nb-2Cr (at%) is an important alloy with exceptional properties, enabling the development of lightweight, efficient, and durable components for various industries. Heat treatment (HT) and hot isostatic pressing (HIPing) are crucial processes for optimizing the microstructure and mechanical properties of Ti-48Al-2Nb-2Cr (at%). Heat treatment enhances desired phase formation, strength, and ductility, while HIPing improves density, mechanical properties, and lifespan. Combining these techniques in one process (i.e., HT-HIP) unlocks the full potential of Ti-48Al-2Nb-2Cr (at%) for advanced materials. Thus, this study presents a new approach to address common issues in Ti-48Al-2Nb-2Cr (at%) processed by L-PBF, such as residual stresses, internal defects, and low ductility. The HT-HIP process combines microstructure improvement and part densification into a cost-effective single process. The proposed method is presented as a multicycle process to investigate the influence of each cycle on the part quality and microstructure formation. The in-depth residual stresses, part defects, microstructure evolution, grain structure, and nanohardness are compared with and without the HT-HIP process. This study makes a significant contribution to sectors that prioritize the development of lightweight, efficient, and durable components, such as aerospace, automotive, and energy industries. By specifically examining the effects of HT-HIP on titanium aluminide, this research effectively tackles the persistent challenges of high residual stresses, internal defects, and low ductility commonly observed in laser-melted Ti-48Al-2Nb-2Cr (at%). The findings directly address these issues, providing valuable insights for improving the performance and reliability of TiAl components in demanding industrial applications.

## 2. Materials and Methods

### 2.1. The HT-HIP Process

**Figure 1:**
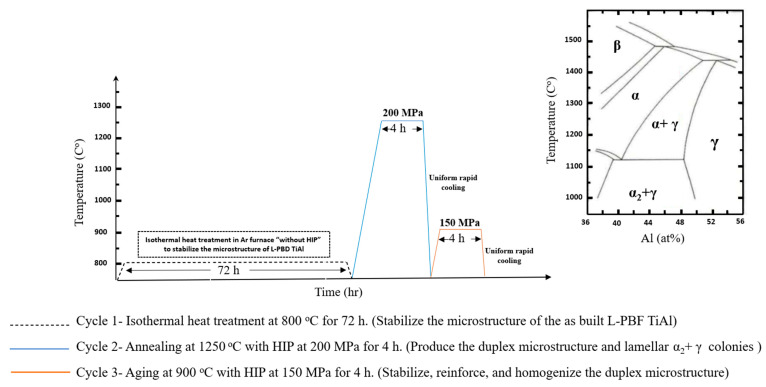
HT-HIP plan.

**Figure 2:**
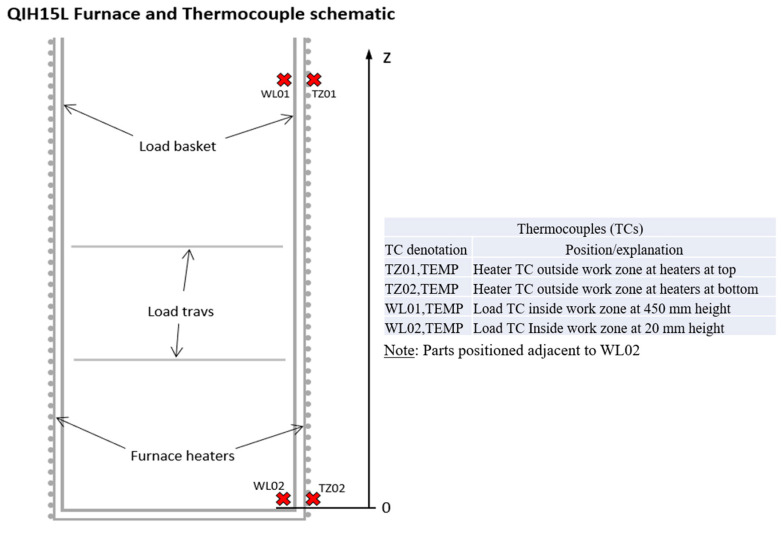
Schematic representation for the QIH15L Moly URC Furnace.

In this study a predefined heat treatment was established along with hot isostatic pressure (HT-HIP) as one process. This combined process was held after processing TiAl by L-PBF under preheating temperature of 200 °C and using sequential thermal scanning (STS) strategy. The HT-HIP included three cycles, as shown in Figure 1. The first cycle was held at 800 °C for 72 h inside an argon furnace to stabilize the microstructure of the as-built TiAl [4]. The second and third cycles were held in QIH15L Molybdenum URC Furnace (Quintus Technologies LLC, Lewis Center, OH, USA), as shown in Figure 2. The QIH15L Molybdenum URC HIP furnace offers a temperature uniformity of ±8 °C or better throughout an operational temperature range of 500–1400 °C, corresponding to furnace class 3 in AMS2750. Type B temperature sensors complying with maximum permitted error according to AMS2750 were used, and the management of the system aligns with practices defined in many industrial standards, such as ASTM A1080. The conditions for the three cycles are presented in Table 1. Annealing at 1250 °C was performed during the second cycle to produce duplex microstructure, while aging at 900 °C occurred during the third cycle to stabilize, reinforce, and homogenize the microstructure. The last two cycles were conducted under high temperature and pressure to generate compressive stresses that help to close micropores and cracks generated during the L-PBF. The heat treatment and isostatic pressure conditions were determined according to previous studies [11,37].

**Figure 3:**
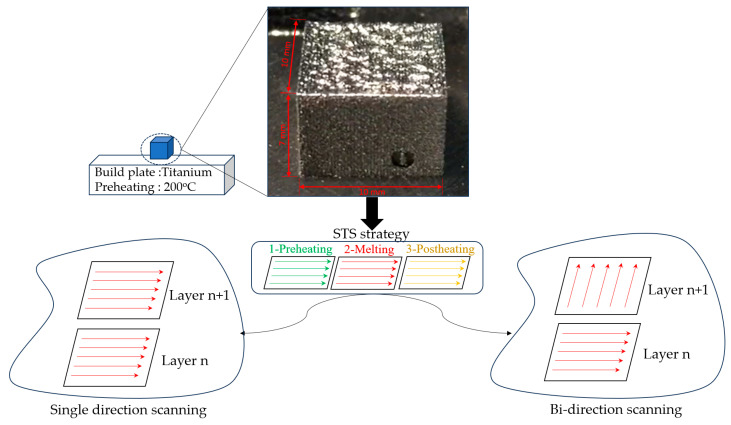
L-PBF sample’s information and conditions.

To study the influence of the HT-HIP process and compare it with the traditional HT method, three TiAl samples (10 × 10 × 7 mm^3^) were fabricated using L-PBF under a preheating temperature of 200 °C and using STS strategy, as shown in Figure 3. One of these samples was applied to the HT-HIP and another was applied to HT. The third sample remained in the as-built condition to be compared with the other two samples. On the other hand, four samples were fabricated to investigate the influence of the HT-HIP cycles. Two of these samples remained in the as-built condition to compare with the other two samples applied to cycle# 2 and cycles #2 and 3. Table 2 shows the TiAl samples designation and the corresponding laser melting and processing conditions for each sample. The fundamental mechanical and thermal properties that can influence the behavior of TiAl during the postprocessing are presented in Table 3.

### 2.2. Evaluation of In-Depth Residual Stresses

**Figure 4:**
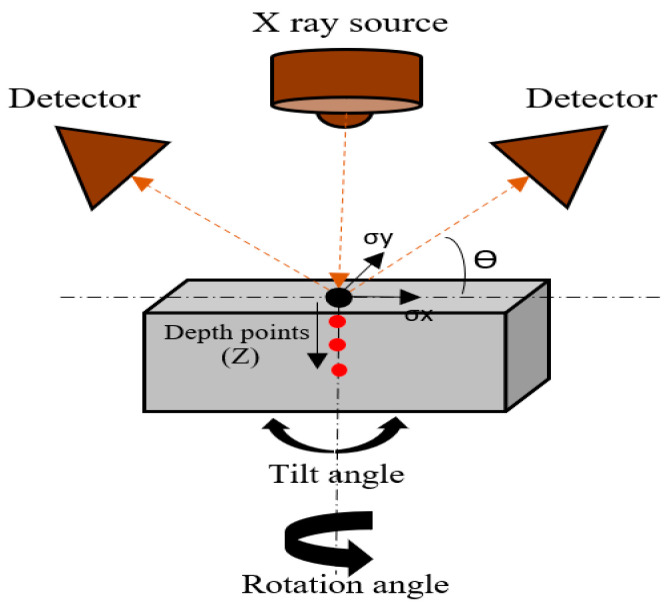
Schematic representation of XRD goniometer used for measuring the residual stress.

The in-depth residual stresses (RS) were measured for the as-built and HT-HIP samples by a high-speed X-ray diffraction (XRD) machine installed with two detectors (Proto Mfg. Ltd., LaSalle, ON, Canada), as shown in Figure 4. The instrument alignment was verified using the ASTM E 915 standard. In XRD technique, the residual stresses are determined by the deformation/strains that occurred in the material crystal lattice as described in [40]. Typically, the internal cracks are caused by high residual stresses induced during the L-PBF process [41]. Therefore, the impact of the HT-HIP method on mitigating these stresses was studied. The in-depth RS was measured on samples fabricated using the same laser process parameters to evaluate the effect of the postprocessing. The residual stresses were measured along ~1 mm depth (34 layers). A Cu K_α_ target was used to diffract from the (213) plane at a diffraction angle (2ϴ) of approximately 149°. The X-ray was targeted to the midpoint of the surface to measure the bidirectional RS. No surface preparation was applied for surface residual stress measurements. Then, the samples were electropolished each time before measuring the depth points to avoid the addition of thermal and/or mechanical stresses. A solution of perchloric acid and methanol was used for electropolishing. The X-ray diffraction residual stress measurements were collected using the multiple exposure technique with a minimum of 22 angles (titling angles) up to 25°. A Gaussian function was used to fit the diffraction peaks. The residual stress measurements were performed using the Proto LXRD stress analyzer.

### 2.3. Defects Investigation

The surface and internal defects such as pores and cracks were investigated for the as-built and postprocessed samples shown in Table 2. The samples were cut vertically, parallel to the building direction, at different locations. The cut sections were mounted, then ground and polished (i.e., up to polishing pad of 3 µm, then MD-Chem with oxide polishing suspension OP-S) according to the ASTM E3-11. The polished surfaces were investigated against defects using Keyence VH-ZST optical microscope (Keyence, Itasca, IL, USA).

### 2.4. Microstructure Investigation

The microstructure evolution was investigated for the different processed samples using a scanning electron microscope (SEM), TESCAN VEGA SEM (TESCAN, Brno, Czech Republic). The samples were scanned using the backscattered electron (BSE) mode to distinguish the different phases. Powder diffraction analysis was applied to identify the phases formed in the 3-cycle HT-HIP sample using a Proto AXRD Benchtop powder diffractometer (Proto Mfg. Ltd., LaSalle, ON, Canada). The data were collected over a wide 2θ range (20°–110°) using Co K-α radiation. The instrument was operated at 35 kV and 30 mA, with a step size of 0.015°, and a dwell time of 8–10 s. In addition, the grain structure was studied using electron backscattered diffraction (EBSD) microscope, FEI Versa 3D Dual Beam SEM/FIB (Thermo Fisher Scientific, Waltham, MA, USA). It was necessary to polish the samples very finely up to 1 µm and then apply OP-S to obtain high-resolution EBSD imaging. TSL OIM Analysis 7 (Version 7.3.1x64, year 2017) (AMETEK, Berwyn, PA, USA) and AztecCrystal software (Version 2.2.302, year 2022) (Oxford Instruments, Oxford, UK) were used to interpret the EBSD data and analyze the grain size, orientation, dislocation density, and angle grain boundaries. The phase formation in the as-built and HT-HIP sample were also identified using EBSD phase mapping.

### 2.5. Nanoindentation Measuring (Nanohardness and Reduced Elastic Modulus)

**Figure 5:**
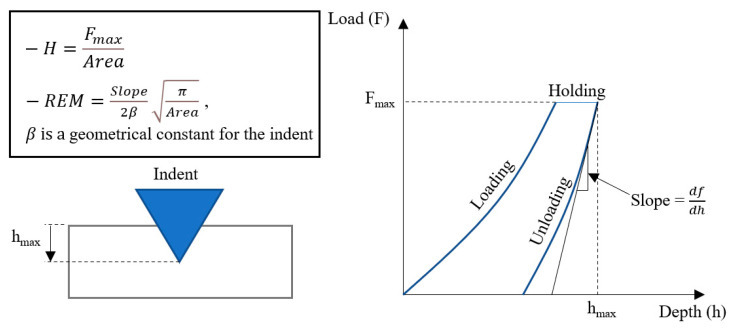
Representative scheme for calculating the nanohardness and reduced elastic modulus by Oliver and Pharr method.

The nanoindentation test was performed using Anton Paar NHT3 tester (Graz, Austria). Nanoindentation is typically used to evaluate mechanical properties (e.g., hardness and elastic modulus) at the nanoscale. The results are typically more accurate than the macro- and microtests. Nanoindentation is essential when measuring the multiphase material of fine microstructures, as is the case in the HT-HIP samples. A Berkovich tip with a pyramidal shape was used for making the indentation. The as-built and HT-HIP samples were mounted and polished to be tested on their cross-section surface. The maximum applied load was 15 mN. The indenter was held for two seconds before unloading. The loading/unloading rate was 30 mN/min. The nanohardness (H) and reduced elastic modulus (REM) were calculated from the loading–unloading curves, as elucidated in Figure 5 and described in the Oliver and Pharr method [42]. At least 7–8 points were measured at different grains to determine acceptable statistical results.

## 3. Results and Discussion

### 3.1. Residual Stresses

The high residual stresses (RSs) induced inside fabricated parts are the main cause of crack formation and poor mechanical properties. It is well known that if the RSs exceed the yield value, the sample will fail due to crack formation and propagation. In L-PBF, the RSs are mainly dependent on the thermal stresses induced by the rapid heating and cooling after the L-PBF. It has been reported that TiAl parts usually experience high tensile stresses after the L-PBF process [43]. These high RSs cause thermal induced cracks below the ductile to brittle transition temperature [7]. Therefore, it was important to investigate the influence of the proposed HT-HIP on mitigating these RSs.

**Figure 6:**
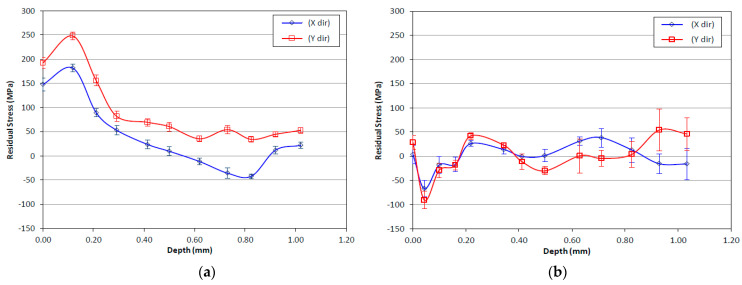
Residual stresses along the sample’s depth: (**a**) as-built-3 sample; (**b**) 3-cycle HT-HIP sample.

**Figure 7:**
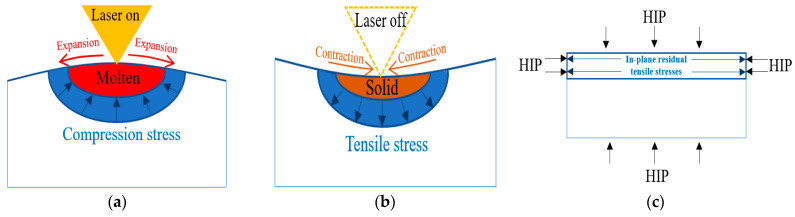
Development of residual stresses in the L-PBF and HT-HIP process: (**a**) L-PBF melting; (**b**) L-PBF solidification; (**c**) HT-HIPing.

The bidirectional RSs were measured along the depth of ~1 mm (34 layers) for two samples, with and without the HT-HIP. Table 4 presents the RS values of the as-built-3 and 3-cycle HT-HIP samples. The data were plotted in Figure 6 to show the RS trend along the depth of the samples. Generally, it was observed that the RSs of the HT-HIP sample were far less than the as-built ones. In addition, the as-built sample showed high tensile RSs up to 248 MPa close to the surface, Figure 6a. The higher RSs in the as-built sample could be attributed to the cyclic thermal expansion and contraction during the melting and solidification of the sample layers in the L-PBF process. As shown in Figure 7, the previously deposited layers compress the newly melted layer against its expansion, while tensioning it against its contraction during solidification. Therefore, tensile residual stresses were induced in the as-built TiAl sample. On the other side, the HT-HIP process could release a big portion of these tensile stresses and introduce compression stresses at different points, as shown in Figure 6b. The mitigation of the tensile stresses could be explained by the application of high-pressure gas perpendicular to the sample surfaces and surrounding all the directions during the HT-HIP process, as shown in Figure 7c. Thus, the resultant RSs in the HT-HIP sample were far less than the as-built RSs. The bidirectional RSs varied between 45 MPa and −90 MPa in the HT-HIP sample, while varied between 248 MPa and −42 MPa in the as-built sample. The tensile RS reduction and the introduction of the compression stresses by the HT-HIP could be beneficial for closing microdefects, as discussed in the coming section.

It was also observed that the RSs in the as-built sample were higher in the X-direction than Y-direction. This agrees with the fact that the thermal gradient is higher in the melt direction than the hatch direction, Y [44]. The high thermal gradient is typically attributed to the rapid cooling that follows the localized laser heating along the melt track. On the contrary, the RSs in the HT-HIP sample showed more stable values in the two directions along the depth. This emphasizes the effect of HT-HIP process to uniform the internal stresses in the sample. However, it was noted that the first few layers showed unstable RSs in both samples. This could be attributed to the surface roughness that affects the accuracy of RSs at the top surface when measured by the XRD method [45,46]. Therefore, the peak RS for the as-built sample, 248 MPa, and the HT-HIP sample, −90 MPa, were observed after a few layers of the top surface. The as-built sample also showed a significant reduction in the tensile stresses along the depth of the sample. This could be explained by the fact that during cooling, the surface layers solidify more rapidly than the subsequent layers, leading to a high contraction at the upper surface [47]. Therefore, high tensile stresses against this contraction were induced in the upper layers and gradually mitigated in the core direction.

The mitigation of tensile residual stresses within the as-built sample can be attributed to the influence of heating the underlying layers during the melting of each successive layer. This heat transfer facilitates the release of residual tensile stresses, resulting in a less stressed core within the sample. Notably, Figure 6a illustrates that the effective depth of this heat transfer is approximately 0.45 µm, or 15 layers, beyond which the residual stress values reach a state of relative stability. This observation highlights the significant role played by laser energy and melting conditions in determining the magnitude and depth of residual stresses within the as-built part. It is important to note that defining the laser energy for the L-PBF can have a substantial impact on the distribution of residual stresses. Higher laser energy levels, for instance, can increase the melting energy of the current layer and lead to a greater heat transfer to the subsequent layers, penetrating to a greater depth compared with lower laser energy settings. This enhanced heat transfer can potentially aid in the release of tensile residual stresses at greater depths within the sample. Consequently, it is recommended for future studies to focus on minimizing residual stresses throughout the depth of the manufactured part by optimizing the process conditions. It is also essential to credit the high reduction in tensile RSs in the as-built sample to the sequential thermal scanning strategy applied by L-PBF, which has been reported to greatly reduce internal defects [14].

In essence, the reduction in residual stresses achieved through the HT-HIP process can be attributed to the high temperature and pressure conditions applied during this postprocessing technique. The elevated temperature during hot isostatic pressing (HIP) facilitates the diffusion of various defects, including voids, dislocations, and porosity, that commonly present in the as-built TiAl parts manufactured using laser powder bed fusion (LPBF). This diffusion process allows the defects to rearrange and heal, resulting in a more uniform microstructure and a reduction in stress concentrations within the material. Moreover, the high pressure exerted during the HIPing process induces compression stresses and plastic deformation in the TiAl material. This plastic deformation enables the redistribution and relaxation of the tensile residual stresses, further contributing to the overall reduction in stress levels within the HT-HIP sample.

It is also worth noting that the deviation of the RSs after applying the HT-HIP increased compared with the as-built sample, Table 4. This could be attributed to more constituent phases formed in the HT-HIP sample, which could be demonstrated in the diffraction pattern and microstructure evolution that is discussed in the following sections.

### 3.2. Part Defects

The main challenge of processing TiAl by L-PBF is the formation of defects such as pores and cracks. These defects are destructive to the part performance under different applications. The pores and cracks are typically classified by the reason of formation. The pores are formed due to lack of fusion, unmolten powder, keyhole mode, and/or gas retention. However, the cracks are typically formed due to solidification shrinkage, elemental segregation, and/or thermal induced stresses. More details about these defects and their causes have been discussed in [48]. The laser process parameters were demonstrated to control the defects in different alloys [49,50,51]. However, the defects in intermetallic material such as TiAl were hard to be controlled by only the laser parameters. Therefore, postprocessing via HIPing was highly recommended. As reported in the literature, there was a direct relationship between the residual stresses and crack formation [43]. Therefore, reducing the residual stresses was a great benefit of the proposed HT-HIP process, as demonstrated in the previous section. Accordingly, the influence of the proposed HT-HIP using the different conditions and cycles, referred to Table 2, were investigated against surface and internal defects.

**Figure 8:**
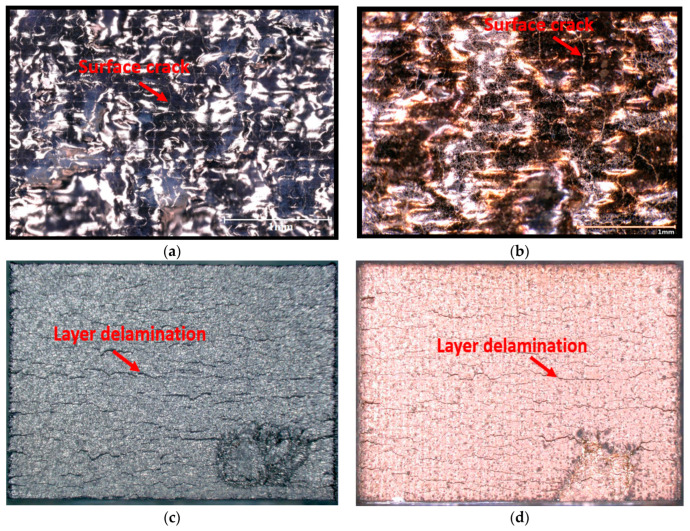
Optical images for the surface defects on the as-built and HT-HIPed samples: (**a**) as-built top surface; (**b**) HT-HIPed top surface; (**c**) as-built side surface; (**d**) HT-HIPed side surface.

**Figure 9:**
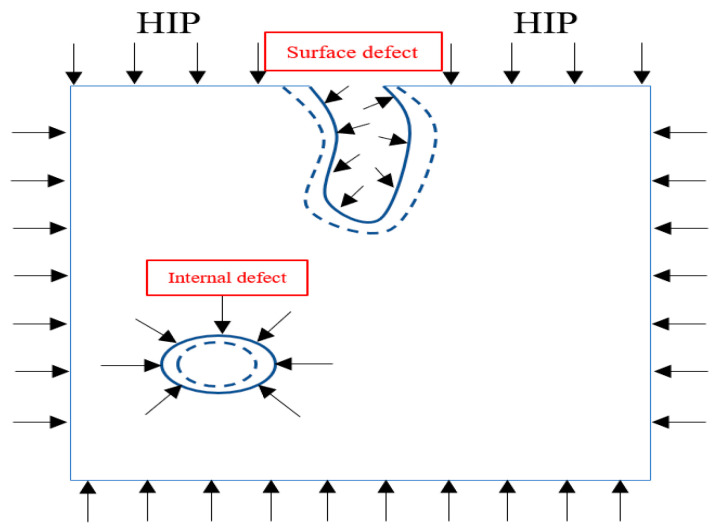
Schematic representation of the HIP effect on surface and internal defects.

**Figure 10:**
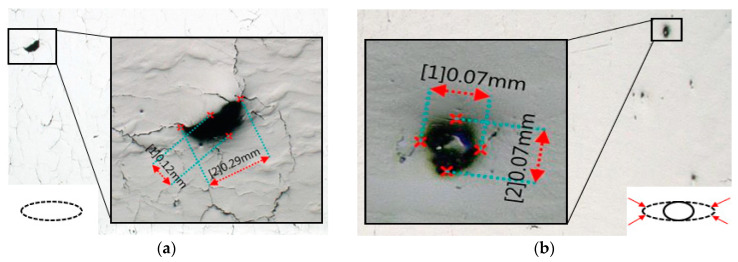
Pore size and shape with and without HT-HIP: (**a**) as-built sample; (**b**) HT-HIPed sample.

The optical examination of surface defects revealed no substantial reduction following the HT-HIP process. This finding was demonstrated by comparing the top and side surfaces of the as-built and HT-HIPed samples, as shown in Figure 8. The optical images clearly revealed the persistent presence of cracks on the top surface even after the HT-HIP process, as indicated by the red arrow Figure 8a,b. Moreover, the delamination layers observed on the side surface of the as-built samples were still apparent following the HT-HIP process, as indicated by the red arrow in Figure 8c,d. To gain insights into these findings, it is crucial to consider the impact of hot isostatic pressure (HIP) on different types of defects. Typically, HIP effectively reduces encapsulated defects (internal defects) by applying pressure on the gap boundaries to minimize their size. In contrast, open defects (surface defects) may undergo swelling due to pressure penetration into the gap. A schematic representation of the effect of HIP on surface and internal defects is provided in Figure 9. Building on this understanding, it is notable that the HIP process significantly influenced the size and shape of internal pores. Figure 10 displays the largest pore size detected in the as-built-2 and 2-cycle HT-HIP samples. Notably, the pore size was substantially smaller and transformed into a regular spherical shape in the HT-HIP sample. This can be explained by the fact that under high temperature and pressure, the material first tends to adhere at the lower radius corners (i.e., pore neck points), and thus, smaller regular spherical pores are formed. Therefore, the combination of optical observations and pore analysis highlights the weak impact of HIP on surface defects, while demonstrating its effectiveness in reducing internal defects and transforming their shape.

**Figure 11:**
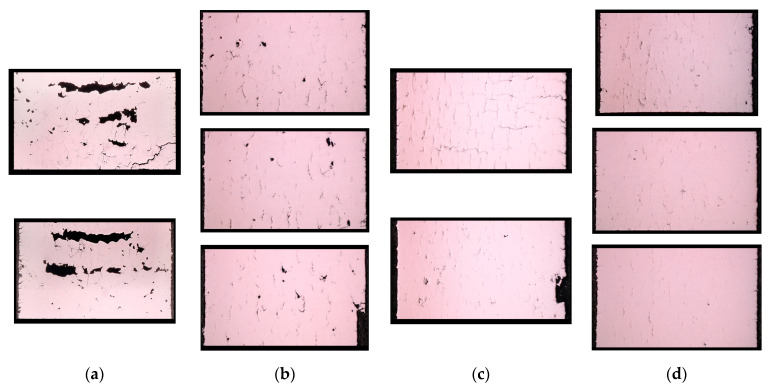
Optical micrographs for the internal defects: (**a**) as-built-1; (**b**) 1-cycle HT-HIP; (**c**) as-built-2; (**d**) 2-cycle HT-HIP.

Figure 11 shows the optical images of full sections in the as-built and postprocessed samples. These sections were cut vertically, parallel to the build direction, at different locations to investigate the existence of micropores and cracks inside the samples. As shown in Figure 11a,b, the pores and cracks greatly reduced in the 1-cycle HT-HIP sample compared with the as-built-1 sample. This reflects the significant effect of the combination of an annealing temperature of 1250 °C and an isostatic pressure of 200 MPa to reduce internal defects. This effect was mainly attributed to the release of residual tensile stresses by applying high temperature, close to melting, and introducing high compressive pressure, higher than the yield stress [36,52]. Figure 11c,d represents the microimaging showing the internal defects in the as-built-2 sample and 2-cycle HT-HIP sample, respectively. It can be observed that the two samples are less internally defected compared with the previous two samples. This refers to two factors: the first is the effect of laser scanning strategy, and the second is the effect of the number of applied cycles. The laser scanning strategy used for fabricating the as-built-1 sample was single-directional laser tracking, while it was bidirectional tracking for the as-built-2 sample. It has been reported that the bidirectional scanning strategy melts the part layers more efficiently than single-directional scanning [11]. Therefore, the pores could be reduced as shown in Figure 11c compared with Figure 11a. The second factor is that the 2-cycle HT-HIP could diminish the cracks and pores compared with the as-built L-PBF and 1-cycle HT-HIP, as shown in Figure 11a–d. This could be attributed to the beneficial double effect of the high temperature and high pressure on reducing internal defects along two cycles. However, it is worth mentioning that the aging cycle at 900 °C and 150 MPa did not show the same high influence on the defects as the annealing cycle at 1250 °C and 200 MPa could. This could be observed by comparing 2-cycle HT-HIP and 1-cycle HT-HIP samples with the as-built-2 and as-built-1 samples, respectively. This is mainly attributed to the lower applied temperature and pressure in the aging cycle compared with the annealing cycle. The relationship between the defect size and effective pressure is governed by Equation (3) [53]. As derived from the equation, the effective pressure (*P*) is increased as the defect radius (*r*) decreases. Knowing that (*γ*) is the specific surface energy, the large volume defects would therefore have been expected to shrink exponentially at low pressure, whereas as they decreased in size, a very high pressure would require their complete closure. Therefore, it can be concluded that the temperature and pressure values are essential factors for the HT-HIP process. However, the temperature factor must be carefully selected but not at the expense of the target microstructure, as discussed in the coming section.

(3) $$P=\frac{2\gamma }{r}$$

**Figure 12:**
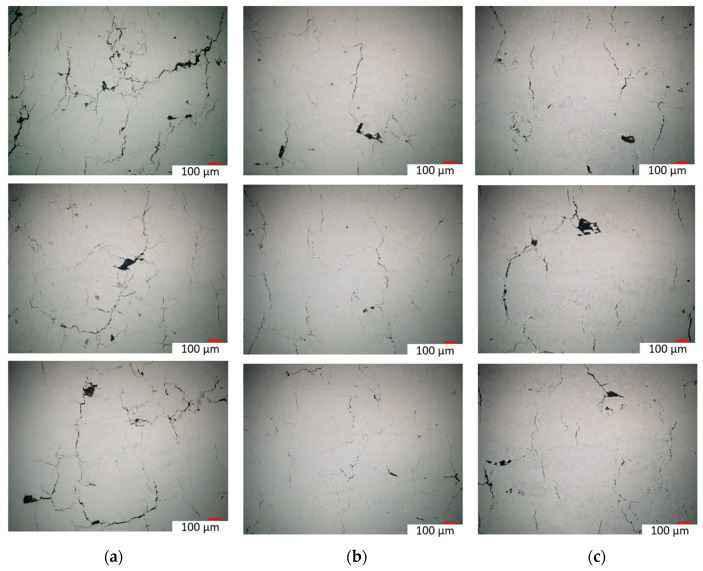
High-magnification optical micrographs for the internal defects: (**a**) as-built-3; (**b**) 3-cycle HT-HIP; (**c**) 3-cycle HT.

Figure 12 shows the high-magnification microimages of three samples with different postprocessing conditions: as-built-3,3-cycle HT-HIP, and 3-cycle HT samples. The three samples were laser-processed at higher volumetric energy density compared with the previous samples. It was demonstrated that the laser energy density affects the internal defect formation. More microcracks were observed in the as-built-3 sample compared with as-built-2 sample. This indicates that increasing the laser energy beyond the optimum can cause overheating and induce microcracks. Alternatively, the effect of the proposed HT-HIP postprocess on the internal defects was compared with the postheat treatment (HT) process, as shown in Figure 12b,c. It was obvious that the isostatic pressure was essential to reduce the internal defects, as shown in the 3-cycle HT-HIP sample, while the HT sample with no pressure showed more defects. This confirms that high temperature can enhance the adhesion of the material, but the isostatic pressure is also necessary to compress/densify the material particles. This in turn led to defect a reduction in all HT-HIP samples. In general, the multiple-cycle HT-HIP postprocess showed a significant reduction in the internal defects of L-PBF TiAl samples. However, it can be concluded that at a certain limit of defect size, increasing pressure rather than repeating cycles can be beneficial to diminish small defects, referring to Equation (5).

Defect reduction or elimination via the HT-HIP process can be explained on a microscale by the simultaneous effects of both the material diffusion and transportation mechanisms that can occur under hot isostatic pressure [36]. During the diffusion mechanism, defect gaps get filled by grain boundary and lattice dislocations, with the magnitude of the isostatic pressure applied playing a vital role in increasing the crosslinking and density of these dislocations. The result is an increased densification level and a concomitant decrease in average defect size. By way of contrast to the diffusion mechanism, the applied pressure also causes surface movement from one point to another on defect boundaries—this is called the surface transportation mechanism. As mentioned above, the preferred path for this mechanism is to start from defect neck points to build up a uniform defect shape at the end. Therefore, the transportation mechanism leads to defect reshaping rather than densification. The results of these two mechanisms are thus a reduction in the defect size and the formation of regular defect shapes that can resist fatigue stresses for longer periods.

### 3.3. Microstructure Evolution

**Figure 13:**
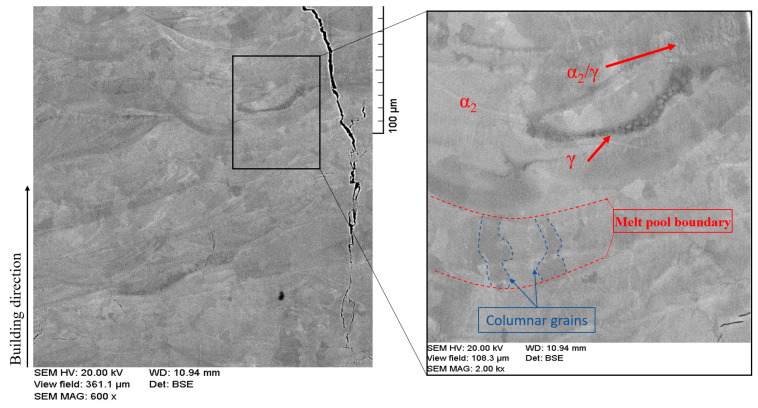
BSE-SEM image for the as-built microstructure.

Processing the microstructure is of great importance for improving material behavior. Grain shape, size, and phase formation are vital factors when controlling the toughness of TiAl parts [54]. It has been reported that Ti-48Al-2Cr-2Nb (at%) parts fabricated by the L-PBF have high strength but low ductility [2]. The reason behind the low ductility is the rapid heating and cooling that freezes the high-temperature phase, α phase, and does not allow the more ductile phase, γ phase, to significantly appear and grow in the microstructure [20]. Thus, the dominant phase in the TiAl parts fabricated by L-PBF was α_2_ [14]. The α_2_ phase is the long-range hexagonal-ordered structure that forms below the eutectic point from the disordered α phase. The high strength but poor ductility are characteristics of α_2_ phase. Figure 13 shows the as-built microstructure of the L-PBF sample fabricated at volumetric energy density of 101.01 J/mm^3^. It can be seen in the figure that the dominant phase was the light gray α_2_ phase, while a scarce amount of dark gray γ phase was also noted. The dark gray areas noticed along and just below the melt pool boundaries were presumably formed due to the remelting effect and the lower temperature gradient that might allow the Al-rich grains (γ phase) to grow and appear in these spots. A small portion of nearly lamellar α_2_/γ was also observed, referred to by the red arrow. This agrees with the published studies about the as-built L-PBF microstructure [8,9,34]. The melt pool boundaries were easily distinguished in the as-built sample, referred to by the dashed red lines. These boundaries usually act as grain nucleation sites. Therefore, the growth of some elongated (i.e., columnar) grains could be observed perpendicular to the melt pool boundary and parallel to the building direction, referred to by the dashed blue lines.

**Figure 14:**
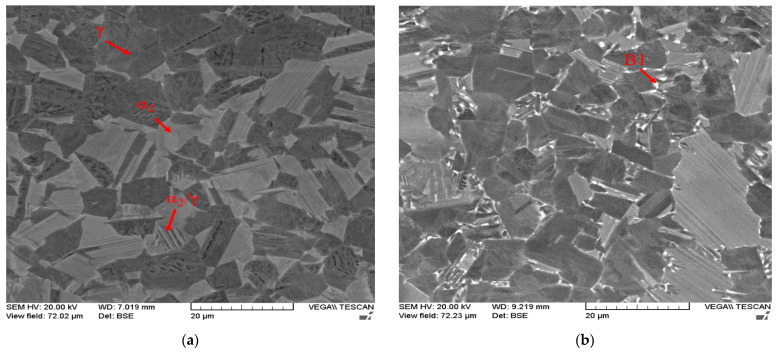

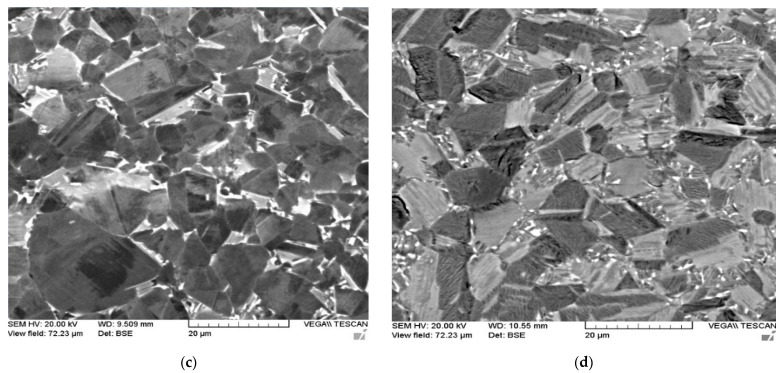
BSE-SEM images for the samples after different postprocessing cycles: (**a**) 1-cycle HT-HIP; (**b**) 2-cycle HT-HIP; (**c**) 3-cycle HT-HIP; (**d**) 3-cycle HT.

The effect of the different postprocesses and number of cycles on the microstructure was investigated. Figure 14 shows the microstructure evolution for four postprocessed samples: 1-cycle HT-HIP, 2-cycle HT-HIP, 3-cycle HT-HIP, and 3-cycle HT. Figure 14a shows the formation of duplex microstructure in form of α_2_ and γ grains after 1-cycle HT-HIP. Lamellar α_2_/γ colonies were also observed. This emphasizes that the annealing temperature at 1250 °C, in the α + γ region, could allow the small nucleus of γ to grow and appear significantly with α_2_ in a dual-phase microstructure. In addition, holding the annealing temperature for 4 h gave rise for the γ grains to diffuse with the α_2_ in lamellar colonies. Finally, applying uniform rapid cooling (URC) could retain this microstructure at room temperature. The transformation from α to lamellar α+ γ is governed by the nucleation and growth process. Nucleation is typically preferred at the grain boundaries and occurs during the formation of the planar faults in the parent α phase [55,56]. Therefore, the lamellar size is dictated by the nucleation sites density. As mentioned before, this duplex microstructure is favored to increase the ductility of TiAl parts. Figure 14b shows the microstructure of 2-cycle HT-HIP sample. It can be demonstrated that the second cycle, the aging cycle, at 900 °C for 4 h, boosted the existence of the lamellar colonies in the microstructure by giving more time for the growth and diffusion between the two phases. It was also observed that few Nb-rich precipitates formed at the grain boundaries, represented by the white B1 phase. The B1 phase was reported as the retained β phase [57]. This phase typically forms due to the precipitation of β stabilizer elements such as Nb or Cr. The main characteristic of B1 is the high hardness. Therefore, a comparison of the microstructure of 1-cycle and 2-cycle samples indicates a trade-off between the strength, ductility, and hardness of the samples. Typically, the duplex microstructure is favored for better ductility. Increasing the cycles promotes the lamellar colonies for better strength. However, the Nb-rich precipitates increase the hardness, which may hinder the ductility of the sample.

**Figure 15:**
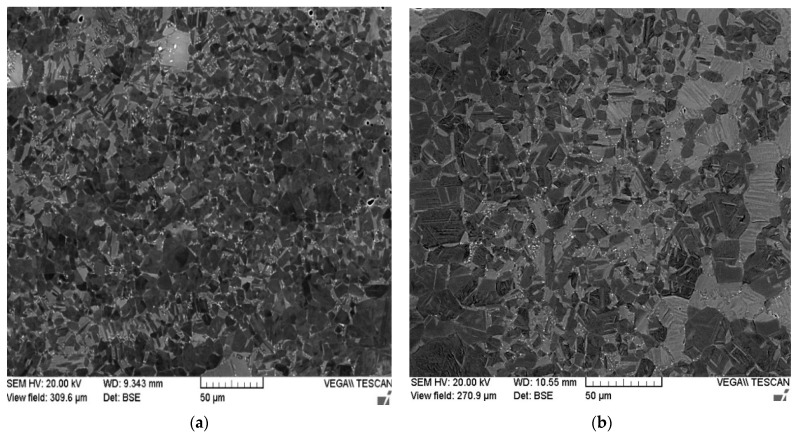
Low-magnification BSE-SEM images for (**a**) 3-cycle HT-HIP and (**b**) 3-cycle HT samples.

**Figure 16:**
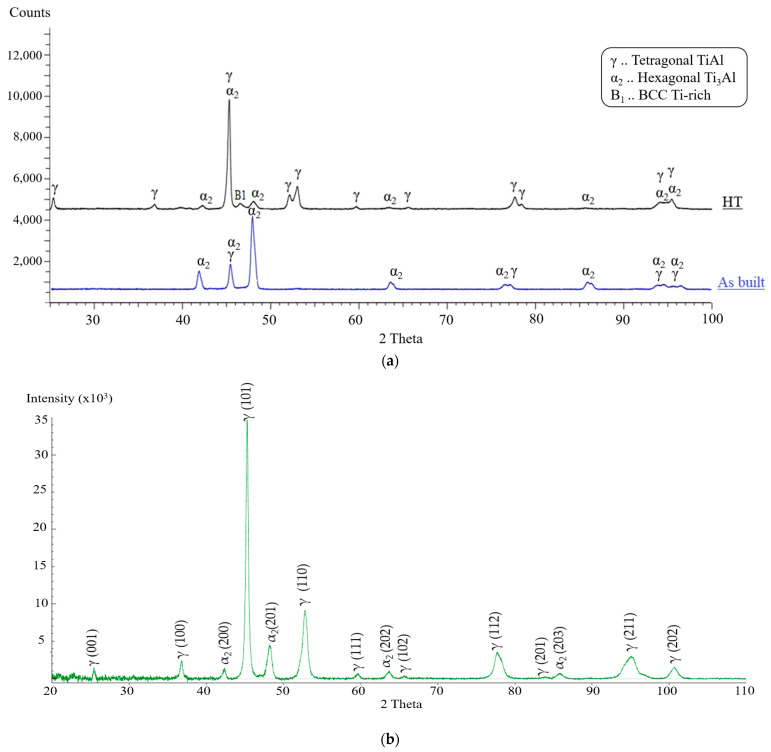
Phase ID for (**a**) the as-built, HT, and (**b**) HT-HIP samples.

The benefit of the proposed HT-HIP is to combine the thermal advantage of the heat treatment to control the microstructure with the mechanical advantage of the HIP process to reduce internal defects. Therefore, it was important to compare the microstructure of a sample postprocessed by HT-HIP with another sample postprocessed by only HT using the same number of cycles. Figure 14c,d shows a duplex microstructure with lamellar colonies for the two samples processed by 3-cycle HT-HIP and 3-cycle HT. Also, the B1 phase was observed in the two samples. However, γ phase can be noted as abundant in the 3-cycle HT-HIP sample. This refers to the more ductile microstructure of the 3-cycle HT-HIP sample. The reason behind this is the longer first cycle for the HT-HIP sample. It is worth mentioning that the first isothermal heat treatment was 72 h for the 3-cycle HT-HIP sample, while it was 24 h for the 3-cycle HT sample. Thus, more γ dendrites could be formed in the first cycle and, consequently, more homogenous duplex microstructure could be achieved in the 3-cycle HT-HIP sample, as shown in Figure 15. The phase formation in the as-built, 3-cycle HT, and 3-cycle HT-HIP samples were identified using the XRD method. Figure 16 shows the phase identification (ID) of the 3-cycle HT-HIP sample, together with published phase IDs for the as-built and 3-cycle HT samples from a previous study [11]. It was determined that the XRD data obtained confirmed the findings obtained via BSE-SEM (See Figure 14). However, it was difficult to clearly detect B1 peaks in the 3-cycle HT-HIP due it is very low volume fraction in the sample. Finally, it can be concluded that the HT-HIP could tailor a similar microstructure to traditional HT but more homogenous and ductile. It can also be concluded that the time and number of cycles played a vital role in controlling the homogeneity and phase formation. Therefore, strength, ductility, and hardness can be tailored along the HT-HIP cycles to meet the required application of the processed part.

### 3.4. Grain Structure

**Figure 17:**
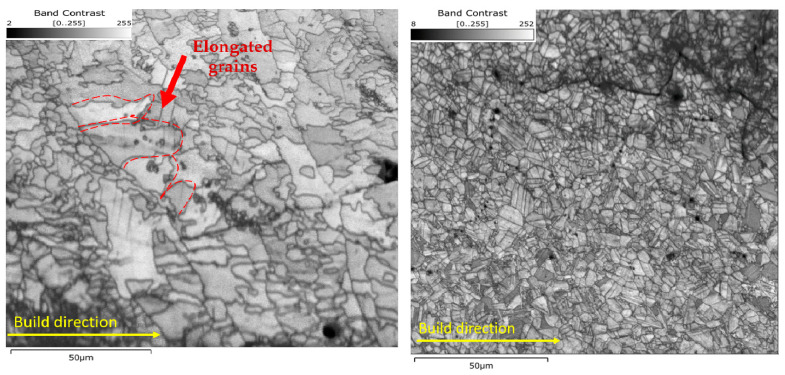

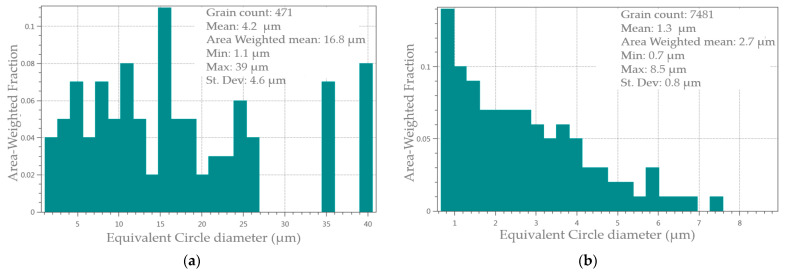
The grain structure and size for (**a**) as-built-2 and (**b**) 2-cycle HT-HIP samples.

The grain structure was studied for two samples with different postprocessing conditions using EBSD analysis. Figure 17 shows the band contrast images and grain size charts of the as-built-2 and 2-cycle HT-HIP samples. In the as-built sample, it can be shown that the grain structure is mainly elongated (i.e., columnar) and parallel to the build direction. This refers to the low thermal gradient (*G*) and high growth velocity (*V*) during the L-PBF. This could be attributed to the sequential thermal scanning, which decreases the thermal gradient, as well as the remelting effect, which increases the grain growth rate. It has been well established that the ratio between the thermal gradient and growth velocity (*G/V*) controls the grain morphology [58,59]. Equation (4) explains the relationship between *G^n^/V* and grain morphology [60,61], in which *G* is the thermal gradient, *V* is the growth velocity, *N_o_* is the nucleation sites/density, ∅ is the volume fraction of the equiaxed grains in front of the solid/liquid interface, and α and n are material constants. According to the equation, the low value of *G^n^/V* indicates the low volume fraction of equiaxed grains in front of the solid/liquid interface (i.e., ∅ < 0.0066). Thus, fully columnar grains are formed in the microstructure. Conversely, the high value of *G^n^/V* indicates the high volume fraction of equiaxed grains (i.e., ∅ > 0.46). Thus, fully equiaxed grains are formed. In the as-built sample, the columnar grains are apparently dominant, but equiaxed grains are still observed. This indicates that the volume fraction of equiaxed grains in front of the solid/liquid interface was close to 0.0066. Conversely, the HT-HIPed sample showed a fully equiaxed grain structure, which indicates ∅ > 0.49. The refined grain structure observed in the HT-HIP sample can be explained by the fact that the grain growth was hindered by the large dislocations formed during the application of the hot isostatic pressure [34]. Typically, these large dislocations combine and accumulate at the grain boundaries, which inhibits the grain growth. Therefore, the nucleation of new grains was favored (i.e., *N_o_* increased) in the HT-HIPed microstructure. It is also noteworthy that the grain size charts indicate coarse grains in the as-built sample with a maximum size of 39 µm, while fine grains in the HT-HIPed sample have a maximum size of 8.5 µm. Twinning boundaries were also observed in the HT-HIPed sample. This was attributed to the atomic displacement occurred during the plastic deformation associated with the hot isostatic pressure. The relationship between the grain size (*d*), twin spacing (*t*), and material strength (*σ_y_*) can be demonstrated using the Hall–Petch equation. As indicated in Equation (5), the material yield strength (*σ_y_*) increases as the effective barrier spacing (*L_eff_*) decreases. The effective barrier spacing is directly proportional to the grain size and twin spacing, as shown in Equation (6) [62]. Thus, both the refined grains and twinning boundaries shown in the HT-HIPed sample demonstrate better strength than the as-built sample.

(4) $$\frac{G^{n}}{V}=\alpha {.\left[\sqrt[{3}]{\frac{-4\pi N_{o}}{3\ln\left(1-\emptyset \right)}.\frac{1}{n+1}}\right]}^{n}=\left\{\begin{array}{c} K_{c},\;\emptyset <0.0066\;\\ K_{e},\;\emptyset >\;\;\;\;0.49 \end{array}\right.$$

(5) $$\sigma_{y}=\sigma_{0}+\frac{K_{y}}{\sqrt{L_{eff}}},\;\mathrm{where}\;\sigma_{0}\;\mathrm{is}\;\mathrm{the}\;\mathrm{dislocation}\;\mathrm{stress}\;\mathrm{and}\;K_{y}\;\mathrm{is}\;H-P\;\mathrm{coefficient}.$$

(6) $$L_{eff}=d.\left[1-\frac{t}{d}+{\left(\frac{t}{d}\right)}^{2}\right],\;\mathrm{if}\;\mathrm{the}\;\mathrm{twin}\;\mathrm{boundries}\;\mathrm{are}\;\mathrm{absent}\;\mathrm{then}\;L_{eff}=d.$$

**Figure 18:**
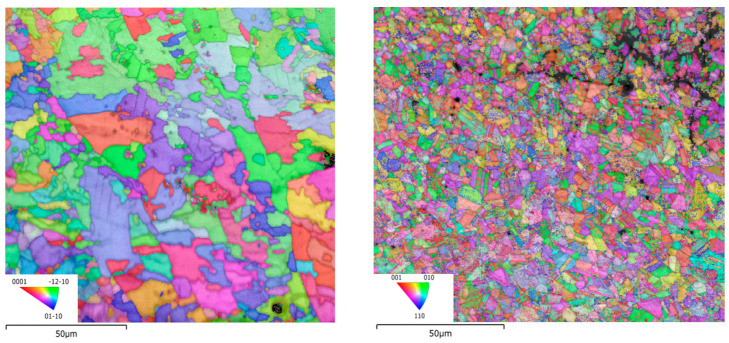

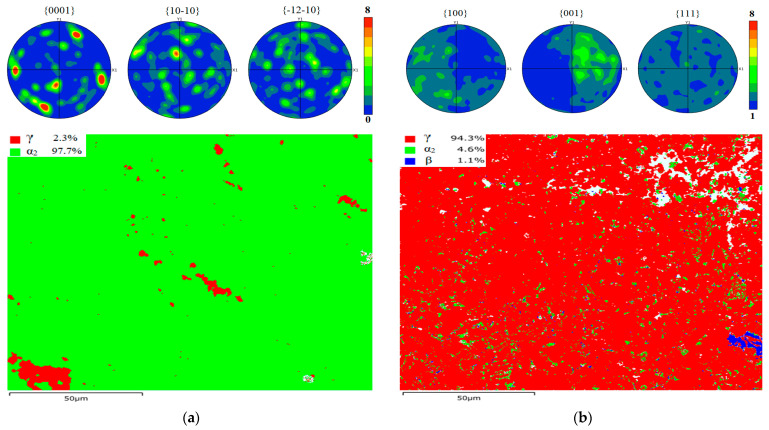
The grain orientation and phase mapping for (**a**) as-built-2 and (**b**) 2-cycle HT-HIP samples.

The grain orientation and phase formation in the two samples was investigated. Figure 18 shows the grain orientation maps, pole figures, and phase maps of the as-built-2 and 2-cycle HT-HIP samples. The orientation distribution was calculated for the main phase of each sample. It could be observed that the as-built grain orientation tends to {0001} direction. This could be attributed to the layer-based nature of the L-PBF, which influences the grains to be oriented parallel to the build direction [48]. On the other side, the HT-HIPed sample did not show a strong tendency for a particular direction. This refers to the more homogenous and isotropic microstructure after the HT-HIP process. It was also noticed that the dominant phase in the as-built sample was α_2_ with 97.7% of the scanned area. In contrast, the dominant phase in the HT-HIPed sample was γ with 94.4%. The α_2_ and retained beta (B1) phases were also observed in the HT-HIPed sample in low proportions of 4.6% and 1.1%, respectively. The demonstration of these formed phases in the as-built and HT-HIPed samples emphasizes the microstructure analysis discussed in the previous section. It is also worth mentioning that the voids and cracks were excluded from the EBSD analysis (indicated by the black and white dots and lines). However, it was necessary to investigate what kind of crack appeared. The band contrast and grain orientation maps demonstrated transgranular crack type. This indicates that the cracks were formed after the grain formation due to thermal-induced residual stresses.

**Figure 19:**
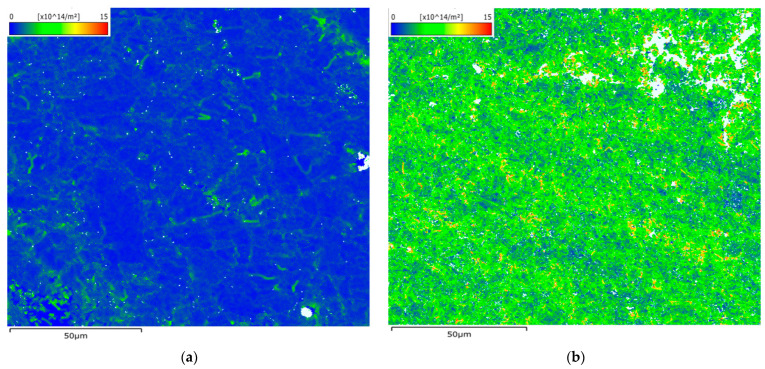
The geometrically necessary dislocation (GND) maps for (**a**) as-built-2 and (**b**) 2-cycle HT-HIP samples.

Geometrically necessary dislocations (GNDs) are a type of dislocation that are concentrated in regions where plastic deformation occurs within a crystal lattice [63]. Unlike statistically stored dislocations (SSDs), which are distributed randomly throughout the material, GNDs are confined to specific areas and play a crucial role in accommodating plastic deformation in materials. In this study, the measurement of GNDs was conducted using a Versa 3D DualBeam SEM/FIB microscope, enabling the observation of crystal lattice distortion and quantification of GND density after hot isostatic pressing (HIPing). The analysis of GND maps was performed using AztecCrystal software. The presence and density of GNDs provide insights into the material flow under stress conditions, specifically the influence of hot isostatic pressure. Figure 19 illustrates that the HT-HIPed sample exhibited a notably high dislocation density. This observation corresponds to the multiple twinning boundaries observed in the band contrast images of the HT-HIPed sample (Figure 17b). The results indicate that the predetermined HT-HIP conditions, including pressure and temperature, effectively induced plastic stress in the sample, promoting material flow to fill voids and cracks. Moreover, the highest dislocation density was typically found around defects, such as cracks and pores in the HT-HIPed sample (indicated by white areas in Figure 19b), indicating significant geometric strain in these areas. Consequently, the reduction in defect size was achieved. Additionally, the high dislocation density observed may also reflect the work hardening effect during the HT-HIP process, contributing to increased strength of the component. Therefore, the analysis of GNDs provides valuable information about the material’s response to the HT-HIP treatment, including the redistribution of dislocations, material flow, defect reduction, and potential strengthening mechanisms.

**Figure 20:**
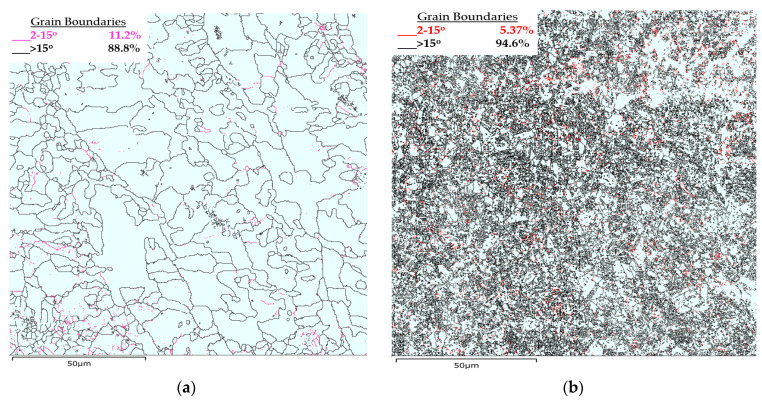
The grain boundary misorientation distribution maps. (**a**) as-built-2 and (**b**) 2-cycle HT-HIP samples.

In L-PBF, grain boundary misorientation angles are typically high (>15°). This is due to the remelting effect, which causes grain recrystallization [64]. Grain residue that cannot undergo recrystallization is typically marked by low-angle grain boundaries (LAGBs). Figure 20 shows the grain boundary misorientation distribution in the as-built-2 and 2-cycle HT-HIP samples. It can be shown that the HAGBs increased to 94.6% in the HT-HIPed sample, compared with 88.8% in the as-built sample. This could be attributed to the grain recrystallization that occurred during the cycles of the HT-HIP process. It can also be observed that the grain boundary density was significantly higher in the HT-HIPed sample than the as-built sample. This indicates a higher dislocation resistance and therefore better strength when the HT-HIP process is applied.

### 3.5. Nanohardness and Reduced Modulus of Elasticity

The nanoindentation test is typically used to determine essential mechanical properties (e.g., hardness, reduced modulus of elasticity, deformation rate, etc.) for delicate microstructure at the nanoscale. It has been reported that the hardness of TiAl samples after post-treatment is phase-dependent [65]. It was also noticed that the postprocessed grains are too small to be measured by microhardness tester. Therefore, the nanoindentation method was used to evaluate the influence of the HT-HIP on the nanohardness (H) and reduced modulus of elasticity (RME) and then to compared it with the as-built nanohardness.

**Figure 21:**
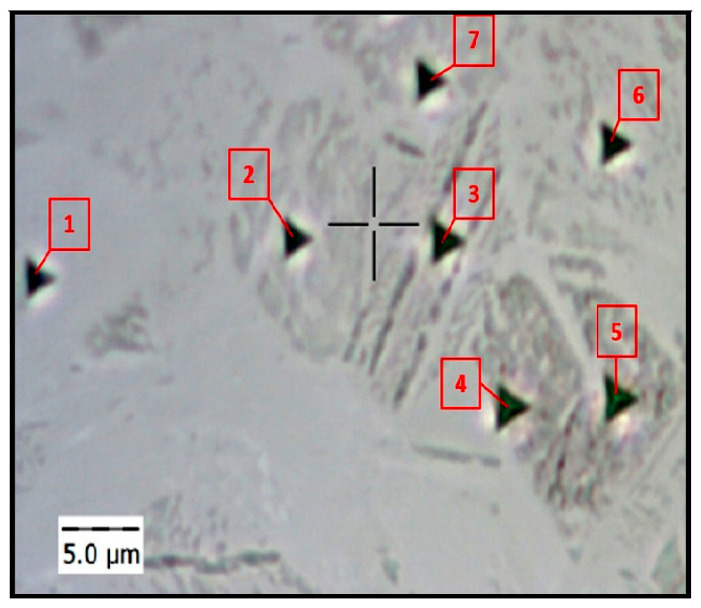
Visual indent pattern at different grains of the HT-HIP sample.

**Figure 22:**
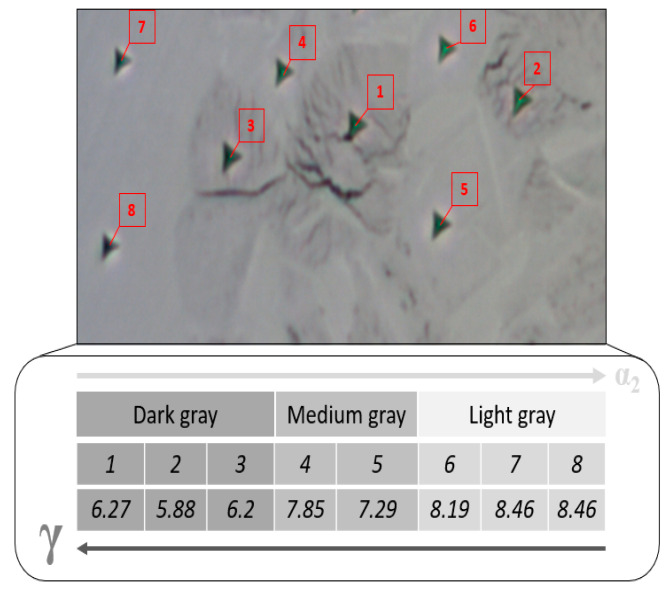
Grain-dependent nanohardness of the HT sample.

As demonstrated earlier, the HT-HIP and HT samples contain α_2_, γ, and β phases. The nanohardness and reduced elastic modulus values have been reported for these phases in the literature [65]. The nanohardness values for the β, α_2_, and γ phases were 7.2 GPa, 5.3 GPa, and 4.2 GPa, respectively. The reduced elastic modulus values were 172 GPa for β, 163 GPa for γ, 149 GPa for α_2_. However, there was a difference in results with other studies [66]. This is attributed to the fact that the nanoindentation values are influenced by the microstructure grain size, lamellar density, and phase proportion inside the grain. Therefore, it was important in this study to measure the nanohardness of the grains rather than the phases. Figure 21 shows seven indentations at different grains of the 2-cycle HT-HIP sample. Table 5 shows the nanohardness (H) and reduced modulus of elasticity (REM) values at different points within the duplex microstructure. It can be noted that the H values varied from 4.28 GPa to 6.39 GPa. This refers to the different phases and lamellar density in the duplex microstructure. For instance, the highest H values at points 1 and 2 correspond to light gray grains in the figure. The light gray color refers to a high fraction of α_2_ phase in the grain. As α_2_ is harder than γ, the nanohardness values were high. On the contrary, the lowest nanohardness values correspond to points 3, 4, and 5. These points are located in dark gray grains, which refer to lamellar-rich structure of γ phase. Thus, the nanohardness values were the least. Between the highest and lowest hardness points, there were medium gray points at 6 and 7. These points refer to close ratios of the α_2_ and γ phases in the lamellar structure with medium nanohardness. Oppositely, the reduced modulus of elasticity showed varied values between 139.4 and 194.2. This agrees to great extent with the calculated and experimental results of the elastic modulus reported in the literature for the α_2_ and γ phases [67]. The same grain-dependent observation of nanoindentation was noted in the HT sample. As shown in Figure 22, the nanohardness values decrease as the lamellar density and γ phase proportion increase inside the grain. The mean nanohardness was 7.32 GPa, which is higher than the nanohardness of the HT-HIP sample. This indirectly refers to the higher toughness of the HT-HIP sample than the HT sample. It is worth mentioning that β-phase grains were too small to be measured accurately by the available instrument. These results demonstrate that the mechanical properties of the duplex microstructure produced by the HT-HIP depend on the phases’ proportion in the lamellar structure inside the grain. Therefore, the number of HT-HIP cycles and their conditions (i.e., temperature and time) play an important role in controlling the final hardness and toughness in L-PBF TiAl.

Table 6 presents the H and REM results of eight points at different grains in the as-built-2 sample. It can be shown that the hardness values were close to each other, with a mean value of 8.19 GPa. This demonstrates that the as-built samples were harder than the HT-HIP samples, which had a mean value of 5.48 GPa. This was attributed to the dominant α_2_ phase in the microstructure of the as-built sample. Meanwhile, the reduced modulus of elasticity showed a mean value of 140, lower than the HT-HIP sample of 168.67. Both the H and REM mean values are close to the α_2_ values reported in the literature, 7.4 GPa and 141 [65,66].

## 4. Conclusions

This study presented a thorough investigation into a novel postprocessing strategy, HT-HIP, for laser-melted Ti-48Al-2Cr-2Nb (at%) alloy. The main objective was to explore the effects of the HT-HIP process on various aspects of Ti-48Al-2Cr-2Nb (at%) samples, including residual stresses, defects, microstructure evolution, and nanohardness.

The HT-HIP process involved three cycles with different conditions. The first cycle employed an isothermal heat treatment at 800 °C for 72 h without applying any pressure. The second cycle encompassed an annealing treatment at 1250 °C for 4 h under a pressure of 200 MPa. Finally, the third cycle consisted of an aging treatment at 900 °C for 4 h under a pressure of 150 MPa.

By implementing the HT-HIP process, significant improvements were observed in terms of residual stresses. The tensile residual stresses, which were prevalent in the as-built samples, were significantly reduced, and compressive residual stresses were introduced in the HT-HIP samples. This adjustment indicated enhanced planar isotropic strength and a notable reduction in internal defects. The size and quantity of defects decreased substantially.

Microstructural analysis revealed noteworthy changes during the HT-HIP cycles. The duplex microstructure consisting of γ and α_2_ phases, along with lamellar colonies, was observed to evolve in varying levels throughout the cycles. Additionally, the presence of the hard B1 phase was detected in the samples subjected to the second and third HT-HIP cycles. The level of duplex microstructure, lamellar colonies, and B1 phase could be controlled through the HT-HIP cycles, allowing for a trade-off between the strength, ductility, and hardness properties. Furthermore, the 3-cycle HT-HIP process achieved a microstructure similar to that achieved with a 3-cycle HT process. However, prolonging the holding time during the isothermal HT cycle in the 3-cycle HT-HIP process could enhance the microstructure homogeneity and the presence of the ductile γ phase.

Grain structure analysis showed that the HT-HIP process leads to significant grain refinement compared with the as-built samples. Furthermore, twinning boundaries were observed in the HT-HIP microstructure, indicating increased strength compared with the as-built counterparts. The orientation of grains along the build direction {0001} observed in the as-built samples was significantly reduced after the HT-HIP process, resulting in a more homogeneous microstructure.

The nanohardness values of the HT-HIPed samples showed dependency on the formed phases and lamellar density. In contrast, the as-built samples exhibited relatively stable nanohardness values due to the dominant α_2_ phase. The variation in nanohardness values of the HT-HIPed samples was attributed to the different levels of duplex microstructure and lamellar colonies within the grains.

In summary, the HT-HIP process demonstrated substantial improvements in terms of the residual stresses, defects, microstructure, and hardness properties of the Ti-48Al-2Cr-2Nb (at%) alloy samples. These findings highlight the potential of HT-HIP as an effective postprocessing strategy for enhancing the performance and reliability of laser-melted Ti-48Al-2Cr-2Nb (at%) components in various industries.

## Figures and Tables

**Table 1:** HT-HIP conditions.

Cycle #	Pressure (MPa)	Temperature (°C)	Hold Time (h)	Medium	Cooling Type
1	-	800	72	Argon	Furnace cooling
2	200	1250	4	Argon	URC
3	150	900	4	Argon	URC

**Table 2:** Experimental plan.

Sample Designation	L-PBF Condition	Processing Condition
As-built-1	Single-directional scanning at E_v_ * = 101.01 J/mm^3^	As-built
1-cycle HT-HIP		Cycle # 2
As built-2	Bidirectional scanning ** at E_v_ = 101.01 J/mm^3^	As-built
2-cycle HT-HIP		Cycles # 2 and 3
As built-3	Bidirectional scanning at E_v_ = 138.89 J/mm^3^	As-built
3-cycle HT-HIP		Cycles # 1, 2, and 3
3-cycle HT		Cycle # 1 (24 h), 2, and 3

* E_v:_ Volumetric energy density. ** Bidirectional scanning: scanning a layer in X-direction, then the next layer in Y-direction.

**Table 3:** γ TiAl mechanical and thermal properties * [38,39].

Density (g/cm^3^)	Yield Tensile Strength (MPa)	Ultimate Tensile Strength (MPa)	Young’s Modulus (GPa)	Elongation to Break	Thermal Expansion Coefficient (×10^−6^ K^−1^) **	Thermal Conductivity (W/m.K)	Melting Point (°C)
3.7–3.9	400–630	450–700	160–176	Up to 3%	9.1	22	1460

* The values differ depending on the processing method and part quality. ** Thermal expansion coefficient is temperature-dependent; this value was reported at 100 °C.

**Table 4:** Residual stress measurements for the as-built-3 and 3-cycle HT-HIP samples.

Depth (mm)	As-Built-3			
	X		Y	
	Residual Stress (MPa)		Residual Stress (MPa)	
0.000	+148	±13	+192	±11
0.118	+182	±8	+248	±8
0.211	+90	±8	+156	±11
0.292	+54	±10	+82	±10
0.414	+24	±9	+70	±8
0.500	+10	±9	+61	±9
0.620	−11	±7	+36	±6
0.730	−35	±10	+54	±8
0.824	−42	±5	+34	±6
0.920	+12	±8	+45	±5
1.020	+22	±7	+53	±6
	3-Cycle HT-HIP			
0.000	+4	±19	+29	±14
0.043	−67	±17	−90	±18
0.099	−18	±17	−29	±15
0.160	−16	±14	−18	±10
0.218	+27	±6	+42	±7
0.343	+14	±9	+23	±6
0.412	0	±6	−11	±16
0.498	+2	±12	−30	±8
0.630	+32	±8	+1	±35
0.711	+38	±19	−4	±17
0.826	+13	±25	+4	±26
0.930	−15	±21	+54	±43
1.034	−16	±32	+46	±34

Note: “+” sign indicates tensile residual stress and “−” sign indicates compressive residual stress.

**Table 5:** Nanohardness and reduced modulus of elasticity at different grains of the HT-HIP sample.

	1	2	3	4	5	6	7	Mean	Std. Dev.
H (GPA)	6.39	6.33	4.98	5.04	4.28	5.61	5.76	5.48	0.77
REM	157.7	175	139.4	194.2	162.9	170.8	180.7	168.67	17.57

**Table 6:** Nanohardness and reduced modulus of elasticity at different grains of the as-built sample.

	1	2	3	4	5	6	7	8	Mean	Std. Dev.
H	8.05	8.16	8.10	8.21	7.82	8.20	8.52	8.43	8.19	0.22
REM	135.4	139.6	147.0	138.4	146.6	133.6	144.4	135.3	140.0	5.3

## Data Availability

Not applicable.
